# Proteomic insights into broiler stress responses to LED lighting: Effects on liver proteome under neutral, cool, and warm spectra

**DOI:** 10.1371/journal.pone.0328279

**Published:** 2025-07-15

**Authors:** Alessio Di Luca, Andrea Ianni, Francesca Bennato, Camillo Martino, Michael Henry, Paula Meleady, Giuseppe Martino

**Affiliations:** 1 Department of Bioscience and Technology for Food Agro-Food and Environmental Technology, University of Teramo, Teramo, Italy; 2 Department of Soil, Plant and Food Sciences (DiSSPA), University of Bari Aldo Moro, Bari, Italy; 3 Department of Veterinary Medicine, University of Perugia, Perugia, Italy; 4 National Institute for Cellular Biotechnology, Dublin City University, Dublin, Ireland; 5 School of Biotechnology, Dublin City University, Dublin, Ireland.; Ain Shams University Faculty of Agriculture, EGYPT

## Abstract

Light plays a critical role in poultry production, influencing broiler behavior, immune function, and growth. Effective lighting management optimizes broiler health and performance, with LED lighting emerging as an energy-efficient alternative to traditional fluorescent lights. However, the molecular impacts of different LED light spectra remain underexplored. This pilot study involved sixteen male broilers (four per treatment group), raised under four different lighting conditions. This study used label-free quantitative proteomics to analyze liver protein expression in broilers (3.30 ± 0.20 kg live weight) raised under Neutral (K = 3300 − 3700), Cool (K = 5500 − 6000), and Warm (K = 3000 − 2500) LED lighting compared to Control neon lights (n = 4 per group). On average, 1,181 proteins were identified, primarily involved in cellular functions, metabolism, regulation, localization, and stress responses. Minimal differences were observed between Neutral and Warm LED lighting and Control lighting, with only six and eight proteins differentially expressed. In contrast, Cool LED lighting resulted in significant changes, with 81 proteins differentially expressed. These differences were statistically significant based on the following criteria: a p-value < 0.05 from ANOVA, at least two matched peptides, and a fold-change in abundance of ≥1.5. Key findings include the up-regulation of ribosome-binding protein 1 and ATP-citrate lyase (ACLY) under Control lighting, linked to stress adaptation. Cool LED lighting triggered up-regulation of proteins associated with aminoacyl-tRNA biosynthesis, suggesting stress-related responses. These results obtained in this pilot study indicate that Neutral and Warm LED lighting positively influence the liver proteome hepatic adaptation in broilers, while Cool LEDs may induce stress-related responses in the liver.

## Introduction

Genetic selection of broilers, nutrition, and improvements in management have enhanced the poultry industry, positively impacting the efficiency of broiler feed conversion and growth performance [1,2]. Light affects animal welfare, growth rate, and production economy. Indeed, light is an essential aspect of poultry production as it plays a pivotal role in the development and functioning of the reproductive system and the growth of birds. Moreover, it is an important factor in controlling many physiological and behavioural processes [3,4]. Light intensity, photoperiod, light source, and the different shades of lights are the main features to consider. While the effect of light intensity and photoperiod have been thoroughly investigated [5–8], additional efforts are needed to better understand the influence of different light sources and the different shades of lights on broilers [9,10].

Artificial light serves as the primary source of illumination for chickens in environmentally controlled chicken houses, making it a crucial element in modern poultry management [11]. Incandescent bulbs have been employed for many years in commercial poultry farms due to their low initial investment costs. However, in more recent times, fluorescent lighting has become the predominant source of light used in broiler houses. Given the high energy consumption associated with these lighting sources and the ongoing rise in energy prices, there is an urgent need for energy-saving strategies, leading to the emergence of various new lighting technologies as potential alternatives [4]. Among the new lighting technologies being developed as potential replacements for incandescent light sources, light-emitting diodes (LEDs) are among the most efficient, durable, and capable of retaining light intensity for considerably longer periods. Moreover, LEDs offer several benefits such as compact size, precise wavelength, minimal heat emission, customizable light intensity and quality, and high efficiency in converting electricity into light [12]. These characteristics make LEDs ideal for promoting chick growth in controlled environments. Several studies have investigated the effect of LED light sources on broiler growth performance, welfare, and meat quality [1,11,13,14]. For instance, Olanrewaju et al. [15,16] investigated the impact of light sources and intensity on the welfare, growth performance, and carcass characteristics of heavy broilers. Their studies, highlighted that LED lights could be a better alternative light source than fluorescent lighting in poultry farm, reducing energy cost without compromising the welfare, growth performance, and carcass characteristics of the broilers. Research into the duration and intensity of light emitted by LEDs has demonstrated a notable impact on the behavior and growth of broilers [11,13]. Specifically, the use of monochromatic yellow light (at 580 nm) has been found to enhance broiler live body weight, growth rate, and feed conversion rate when compared to light from white LEDs or fluorescent lamps [17].

Nevertheless, research on the biochemical and molecular processes influenced by LED lighting remains scarce. Proteins serve as the principal functional molecules within cells, leading nearly all biological and cellular processes. Moreover, the interplay between messenger RNA and protein expression is complex, often lacking direct correlation across most genes and exhibiting significant variability from one protein to another [18,19]. Therefore, a better understanding of how protein abundances vary between different light sources could potentially better explain the biochemical and molecular processes underneath. In the past decade, mass spectrometry-based proteomics has garnered growing interest, droved by advancements in sample preparation techniques, instrumentation, and enhanced protein databases. These developments have facilitated the analysis of numerous proteins within a sample, enabling the study of comprehensive protein dynamics across cells, tissues, and organisms [20]. Different approaches have been developed to compare the entire proteome between different conditions. Among these approaches, quantitative label-free proteomics allows for the relative quantification and identification of thousands of proteins from complex samples [21]. In recent years, this approach has been widely used in animal science as well [22–24]. For instance, Di Luca *et al.* [25] utilized label-free quantitative proteomics to identify proteins correlated with transport stress in pigs or able to discriminate between goat breeds [23]. The liver plays a pivotal role in nutrient metabolism and nutrient repartitioning, influencing many physiological processes that affect feed efficiency, growth, and performance traits in all livestock species [26]. This organ has been successfully utilized as a matrix in several proteomics studies in animal science. For example, using this matrix, Tang *et al*. [27] investigated the heat stress response in broilers using label-free quantitative proteomics, identifying 257 proteins differentially expressed between the control and heat stressed groups. Similarly, the liver proteome of two heavy pig breeds was compared using a similar approach, revealing a total of 25 proteins capable of discriminating between the two pig breeds [28].

Therefore, the objective of the present study, utilizing label-free quantitative proteomics, was to investigate how different light sources including three shades of LED lights (Neutral, Cool, and Warm) and Control neon lights, influence liver protein abundance patterns in broilers, aiming to identify functional relationships linked to these variations.

## Materials and methods

### Ethics statement

In this project, liver samples were collected *post-mortem* from chickens routinely slaughtered on a poultry farm for meat production. The chickens were raised by a local farm and were not sacrificed specifically for this study; instead, the research utilized samples from the normal slaughtering procedures at the end of their growing phase, which marks the end of their productive life for meat production. Therefore, no additional ethical statement was necessary. All animal care procedures were performed in accordance with a research protocol that complies with both Italian and European Community directives [29].

### Sampling

Broiler chickens of the Ross 508 strain (Aviagen Group, Huntsville, AL, USA), totaling 12,000 individuals, were used in this study and randomly assigned to four treatment groups, with 3,000 birds per group. The lighting treatments included three LED conditions, Neutral (3300–3700 K), Warm (2500–3000 K), and Cool (5500–6000 K) and one conventional neon lighting group (Control). These values were selected in alignment with available commercial LED technologies and to reflect the most commonly used practical light settings in poultry production environments. Moreover, these values correspond to manufacturer-defined color temperature outputs of the LED fixtures used in the trial, ensuring consistency and reproducibility. Each treatment group was housed in separate building within the same commercial farm in the Abruzzo region of Italy, with each environmentally controlled building (approcimately 70 m × 10 m, total area 700 m²) dedicated to one lighting treatment and accommodating 3,000 birds (approximately 4.8 birds/m²). This setup ensured complete isolation of light conditions across groups. All birds were reared under identical conditions, with the lighting system being the only variable.

Broilers, were hatched from the same incubator located on the same farm where the study was conducted (i.e., the hatchery and the farm were in the same area). Birds were assigned to their respective lighting groups from day one and exposed to a lighting schedule in compliance with European directives (18 hours of light and 6 hours of darkness). Temperature was maintained at 27/31 °C during the first week and adjusted to 23/26 °C from the second week onward. All birds received the same three-phase diet: starter (days 1–12), grower (days 13–21), and finisher (days 22–48); the chemical composition of the diet is detailed in a previous publication [30].

At the end of the production cycle (day 48; commercial weight: 3.5 ± 0.3 kg), four birds per treatment group (totaling 16 birds) were randomly selected as biological replicates to evaluate the proteomic changes in the liver induced by the different lighting conditions. No replications were included within each treatment group, as this study was designed as a pilot trial aimed at exploring proteomic responses in broilers to different lighting conditions under commercial settings. This approach was necessary due to the logistical complexity and large-scale dimensions of the housing structure, which made physical replication of lighting conditions across multiple pens unfeasible. Additionally, in discovery-based proteomics studies, increased replication without strict environmental control can introduce higher variability, potentially masking biologically relevant signals. Detailed procedures for the study design have been described elsewhere [24,30,31]. To minimize bias, all management practices (housing, feeding, and handling) were kept consistent across groups.

On the day of slaughter, the broilers were electrically stunned and processed at a commercial plant. Each group was scheduled sequentially to ensure traceability. Liver samples were collected immediately post-evisceration in a controlled environment, labeled, and stored at 4°C. Protein extraction was performed on the same day, and the resulting samples (exudate) were stored at –80°C until proteomic analysis.

### Sample preparation for label-free LC-MS/MS analysis

Protein extraction from the exudate of each sample was carried out on the day of slaughter, following the protocol described in Di Luca et al. [25,32]. The protein concentration of all samples was measured in triplicate using the Bio-Rad Protein Assay Kit (Bio-Rad Labs, Hercules, CA, USA), based on the Bradford method with a bovine serum albumin (BSA) standard as detailed in [33].

Four biological replicates per group (one sample from each bird) were subjected to label-free analysis. An equal amount (100 μg) of protein from each sample was reduced and alkylated using dithiothreitol (DTT) and iodoacetamide (IAA; Sigma-Aldrich/Merck, USA), then digested with trypsin following the filter-aided sample preparation (FASP) method [32]. The FASP protocol employs an ultrafiltration device with membrane pores that allow detergents to pass through while retaining proteins due to their larger size [34]. The resulting peptides were purified using C18 spin columns (Thermo Fisher Scientific), dried under vacuum, and reconstituted in 2% acetonitrile with 0.1% trifluoroacetic acid prior to mass spectrometry analysis.

### LC-MS/MS and label-free quantitative differential analysis

Peptides from all samples were collected for Nano LC–MS/MS (liquid chromatography-mass spectrometry) analysis using an Ultimate 3000 RSLCnano system coupled in-line with an Orbitrap Fusion Tribrid™ mass spectrometer (Thermo Scientific, USA).

The raw MS files were merged and processed for protein identification using Proteome Discoverer v.2.2 (Thermo Fisher Scientific, USA). LC-MS proteomics data were then analyzed with Progenesis QI for proteomics v.2.0 (NonLinear Dynamics, Newcastle upon Tyne, UK) by searching against a UniProtKB database containing 32,426 protein sequence entries (downloaded in August 2020), which included proteins from *Gallus gallus* (chicken). After alignment and normalization, statistical comparisons between groups were performed using one-way ANOVA integrated into Progenesis QI. Proteins were considered differentially expressed if they met the following criteria: (i) a p-value < 0.05 from ANOVA, (ii) at least 2 matched peptides, and (iii) a fold-change in abundance of ≥1.5. The MS analysis and protein identification methods and parameters adhere to those described by Di Luca et al. [23].

### Bioinformatics

Proteins identified as statistically differentially abundant in the comparisons (Neutral vs. Control, Warm vs. Control, and Cool vs. Control) were classified using the PANTHER (Protein Analysis Through Evolutionary Relationships) database system, release 14.1 (http://www.pantherdb.org/) [35] with default parameters to obtain biological process classifications.

The functional interpretation of these proteins was performed in Cytoscape (http://www.cytoscape.org/) [36] using the ClueGO plugin (http://www.ici.upmc.fr/cluego/) [37]. Gene enrichment analysis focused on the Gene Ontology (GO) - Biological Process (BP) branch, using data from the November 2019 release. The analysis applied the following parameters: GO hierarchy levels 3–8, a minimum of 2 genes per GO term, at least 2% of input genes associated with each term, a GO term network connectivity (Kappa score) of 0.40, and a right-sided hypergeometric test. These settings were chosen to enhance the functional association process. Additionally, the gene enrichment analysis and the organization of pathway term networks were based on *G. gallus*-specific functional annotations from May 2020, as described previously [23]. GO:BP terms with a Benjamini–Hochberg corrected *p*-value of <0.05 were considered statistically over-represented. A separate pathway enrichment analysis was also performed using the ClueGO plugin with the Kyoto Encyclopedia of Genes and Genomes (KEGG) pathway database from the November 2019 release.

For the in silico protein–protein interaction (PPI) analysis, the STRING (Search Tool for the Retrieval of Interacting Genes/Proteins) database (https://string-db.org/; version 11) was used [38]. Analyses were carried out considering the *G. gallus*-specific interactome, and only interactions with a STRING combined score > 0.7 (high confidence) were retained. Network metrics, including the number of nodes and edges, average node degree (average number of connections), the expected number of edges (if nodes were randomly selected), and the PPI enrichment *p*-value, were calculated. A low PPI enrichment *p*-value indicates that the observed network connectivity is statistically significant.

## Results

### Quantification and statistical analysis of proteins differentially expressed in response to various LED lighting shades

In order to analyze changes in chicken liver proteins in broilers (Ross 508) raised under different shades of LED lighting (Neutral, Warm, Cool, and a Control lighting system), we applied a label-free LC–MS approach to quantify proteins obtained from the chicken liver following centrifugation (exudate). After proteomics analysis, a similar number of proteins were identified across the three comparisons. A total of 1,170 proteins (6,903 peptides) were identified across the eight samples (4 samples from each lighting condition) from broilers raised in Neutral LED lighting and with Control lighting (see S1 Table). For broilers raised in Cool LED lighting and with Control lighting, 1,141 proteins (6,261 peptides) were identified across the eight samples (see S2 Table). Meanwhile, 1,231 proteins (6,437 peptides) were identified across the eight samples for broilers raised in Warm LED lighting and with Control lighting (see S3 Table).

**Fig 1:**
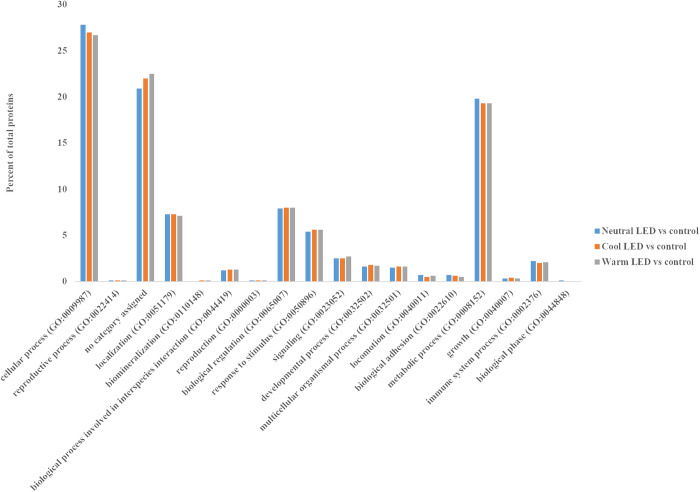
Proteins classification according to their biological processes of all the proteins identified in each comparison (see S1, S2 and S3 Tables).

Gene ontology (GO) analysis of all identified proteins in each comparison was performed using PANTHER, with *G. gallus* genome annotations serving as the background. Fig 1 illustrates the categorization of biological processes for the proteins identified across the comparisons. Proteins identified in each comparison were found to be involved in similar biological processes such as cellular processes, metabolic processes, biological regulation, localization, and response to stimulus (Fig 1).

Progenesis QI for Proteomics was utilized to investigate differences in the proteome across three comparisons. The significance criteria applied were a *p*-value of ≤0.05, fold change of ≥1.5, and proteins with at least ≥2 peptides matched. In the comparison between broilers raised with Neutral LED lighting and Control lighting, six proteins (from 16 peptides) were identified as differentially expressed. Among these, two proteins (33.33%) were up-regulated in the broilers raised with Neutral LED lighting, while four proteins (66.66%) were up-regulated in those raised with the Control, as illustrated in Table 1.

**Table 1:** Birds reared with Neutral LED lightings compared to birds reared with the Control lighting.

UniProt^a^	Gene Name	Description	Peptides^b^	Score^c^	ANOVA(p)	Fold change	Highest condition^d^
A0A1D5PZ32	GBP4L	GB1/RHD3-type G domain-containing protein	4	9.16	0.02	7.34	Neutral LED lightings
A0A1L1RNS0	ENO1	Alpha-enolase	3	10.35	0.023	2.97	Neutral LED lightings
A0A3Q2UM65	RRBP1	Ribosome-binding protein 1	3	7.08	0.026	1.76	Control
A0A3Q2TU25	DPYD	Dihydropyrimidine dehydrogenase [NADP(+)]	2	8.30	0.026	1.74	Control
A0A3Q2U535	DHDH	Dihydrodiol dehydrogenase	2	6.17	0.031	6.29	Control
A0A452J7T4	AP2M1	AP-2 complex subunit mu	2	5.99	0.033	1.89	Control

Six proteins identified with altered levels in response to Neutral LED lightings compared to birds reared with Control following label-free LC-MS/MS analysis (Progenesis QI for proteomics). Two proteins were up-regulated in the birds reared with Neutral LED lightings and 4 proteins were up-regulated in the birds reared with Control lighting.

^a^) Accession number in the UniProt database. ^b^) Peptides used for quantitation. ^c^) SEQUEST score. ^d^) Indicates if the proteins were up-regulated in the birds reared with Neutral LED lightings or with Control lighting.

A larger number of proteins (81 proteins, from 353 peptides) were identified as differentially expressed between broilers raised with Cool LED lighting and the Control lighting. Of these, 30 proteins (37.04%) were up-regulated in broilers raised in Cool LED lighting (Table 2), and 51 proteins (62.96%) were up-regulated in those raised with the Control, as shown in Table 3.

**Table 2:** Proteins up-regulated in birds reared under Cool LED lighting compared to birds reared with the Control lighting.

UniProt^a^	Gene Name	Description	Peptides^b^	Score^c^	ANOVA (p)	Fold change	Highest condition^d^
A0A1D5PE96	PLS3	Plastin 3	15	41.22	0.0023	1.88	Cool LED lightings
F1NQZ9	GCLC	Glutamate-cysteine ligase catalytic subunit	9	24.42	0.0180	2.46	Cool LED lightings
A0A1D5PKV6	XYLB	Xylulokinase	8	23.32	0.0090	2.35	Cool LED lightings
A0A1L1RUF2	RNH1	Ribonuclease/Angiogenin Inhibitor 1	7	24.98	0.0022	2.36	Cool LED lightings
A0A1L1RX59	GARS	Glycine--tRNA ligase	6	16.17	0.0007	1.61	Cool LED lightings
F1NUS2	PREP	Prolyl endopeptidase	6	16.84	0.0041	1.62	Cool LED lightings
F1NJU7	YARS	Tyrosine--tRNA ligase	6	15.15	0.0115	1.88	Cool LED lightings
A0A1D5PX97	PEPD	AMP_N domain-containing protein	5	15.19	0.0031	1.81	Cool LED lightings
Q5ZM75	RCJMB04_2o24	AA_TRNA_LIGASE_II domain-containing protein	4	21.17	0.0081	1.99	Cool LED lightings
A0A1D6UPQ3	TARS	AA_TRNA_LIGASE_II domain-containing protein	4	9.52	0.0274	1.93	Cool LED lightings
A0A1L1RND0	HSP90AA1	Heat shock protein HSP 90-alpha	3	8.33	0.0062	3.63	Cool LED lightings
A0A1D5PTR5	TLN1	Talin-1	3	8.91	0.0093	3.64	Cool LED lightings
A0A1D5NUN3	ACSS2	Acyl-CoA synthetase short chain family member 2	3	8.20	0.0191	2.96	Cool LED lightings
A0A1L1RMC0	PSMA1	Proteasome subunit alpha type	2	6.73	0.0029	1.72	Cool LED lightings
A0A1D5PIZ1	THOP1	Thimet oligopeptidase 1	2	5.99	0.0047	1.74	Cool LED lightings
A0A1D5P901	HAGH	Hydroxyacylglutathione hydrolase, mitochondrial	2	8.68	0.0067	2.08	Cool LED lightings
E1BZY3	GGACT	Gamma-glutamylaminecyclotransferase	2	4.78	0.0072	1.75	Cool LED lightings
A0A3Q2U9P1	FKBP4	Peptidylprolyl isomerase	2	6.59	0.0089	2.55	Cool LED lightings
A0A1D5NYG3	FLNB	Filamin B	2	6.15	0.0103	2.25	Cool LED lightings
F1NT28	PPA1	Pyrophosphatase (inorganic) 1	2	6.35	0.0124	1.88	Cool LED lightings
H9KYX6	SELENBP1	Selenium binding protein 1	2	6.90	0.0145	5.72	Cool LED lightings
E1BQD1	GATD3AL1	Putative glutamine amidotransferase like class 1 domain containing 3A-like1	2	7.50	0.0147	2.76	Cool LED lightings
A0A3Q2TYT7	BCL2L15	BCL2 like 15	2	4.68	0.0150	2.01	Cool LED lightings
Q5ZLD1	FH	Fumarate hydratase, mitochondrial	2	4.62	0.0159	5.93	Cool LED lightings
A0A1D5P470	LOC107050491	Glutathione S-transferase	2	5.34	0.0163	4.93	Cool LED lightings
F1N8A7	RBKS	Ribokinase	2	6.58	0.0199	2.46	Cool LED lightings
F1NCZ2	GDI2	Rab GDP dissociation inhibitor	2	5.82	0.0256	1.89	Cool LED lightings
A0A1L1RJI2	UBE2K	Ubiquitin conjugating enzyme E2 K	2	6.69	0.0305	1.64	Cool LED lightings
A0A1D5PLK6	PDCD6IP	Programmed cell death 6-interacting protein	2	4.71	0.0320	27.37	Cool LED lightings
A0A3Q2UDT6	NPEPPS	Aminopeptidase puromycin sensitive	2	5.38	0.0339	2.20	Cool LED lightings

Thirty proteins up-regulated in birds reared under Cool LED lighting among 81 with altered levels compared to Control lighting, identified by label-free LC-MS/MS (Progenesis QI for proteomics).

^a^) Accession number in the UniProt database. ^b^) Peptides used for quantitation. ^c^) SEQUEST score. ^d^) Indicates if the proteins were up-regulated in the birds reared with Cool LED lightings.

**Table 3:** Proteins up-regulated in birds reared under Control lighting compared to birds reared to Cool LED lightings.

UniProt^a^	Gene Name	Description	Peptides^b^	Score^c^	ANOVA (p)	Fold change	Highest condition^d^
A0A1D5PSE5	ACLY	ATP-citrate synthase	19	44.42	0.0043	2.14	Control
A0A1D5PZJ4	CCT8	T-complex protein 1 subunit theta	17	49.15	0.0003	1.91	Control
Q5F411	CCT5	T-complex protein 1 subunit epsilon	12	30.82	0.0001	2.13	Control
F1NZQ9	HAL	Histidine ammonia-lyase	12	40.98	0.0050	2.17	Control
F1NK38	CCT7	T-complex protein 1 subunit eta	10	30.84	0.0031	2.06	Control
F1NTK1	PSAT1	Phosphoserine aminotransferase	10	36.47	0.0071	1.96	Control
Q5ZMG9	TCP1	T-complex protein 1 subunit alpha	9	29.11	0.0012	2.07	Control
A0A1D5P1D0	PAPSS2	3’-phosphoadenosine 5’-phosphosulfate synthase 2	8	20.58	0.0004	17.84	Control
A0A3Q2UA96	CCT3	T-complex protein 1 subunit gamma	8	21.11	0.0008	2.28	Control
A0A1D5NV94	AP2A2	AP-2 complex subunit alpha	7	17.10	0.0001	2.29	Control
A0A1D5P8N3	AP1B1	Adaptor related protein complex 1 subunit beta 1	7	21.99	0.0004	2.00	Control
Q5F424	CCT2	T-complex protein 1 subunit beta	7	18.80	0.0018	2.23	Control
F1NMC3	MTHFD1	C-1-tetrahydrofolate synthase, cytoplasmic isoform 2	7	16.80	0.0024	1.72	Control
Q9I8D6	tcp-1 delta	T-complex protein 1 subunit delta	6	18.03	0.0007	2.12	Control
A0A1D5NTV3	ACACA	Acetyl-CoA carboxylase 1	6	18.89	0.0251	6.12	Control
A0A452J7Z4	PRPS2	Ribose-phosphate pyrophosphokinase 2	5	15.47	0.0070	2.26	Control
E1C3A9	HDLBP	Vigilin	5	16.29	0.0164	2.93	Control
E6N1V8	GNB2L1	Guanine nucleotide-binding protein beta subunit2-like 1	5	12.74	0.0174	3.74	Control
A7UEA8	GPAT	Amidophosphoribosyltransferase	5	20.02	0.0228	1.76	Control
F1NNV2	ACSL5	AMP-binding domain-containing protein	4	10.76	0.0233	12.98	Control
E1BS37	HSD17B11	estradiol 17-beta-dehydrogenase 11	4	10.68	0.0310	5.26	Control
F1NL38	HRG	Histidine-rich glycoprotein	3	7.22	0.0058	4.95	Control
A0A3Q2UGR5	SEC24C	SC24C protein	3	6.99	0.0058	2.31	Control
Q90XD9	STAT	Transcriptional coactivator p100 (Fragment)	3	9.00	0.0142	2.77	Control
A0A3Q2TV58	LOC112530469	Cytochrome P450	3	11.27	0.0210	8.34	Control
A0A1D5PEI5	SEC24C	Tubulin beta chain	3	7.99	0.0268	1.80	Control
A0A1D5PT58	RPS8	40S ribosomal protein S8	3	9.08	0.0289	3.86	Control
E1BSJ2	RPS21	40S ribosomal protein S21	3	8.44	0.0353	3.22	Control
A0A3Q3A7Y5	CLAPS2	Adaptor related protein complex 2 subunit sigma 1	2	4.64	0.0012	7.07	Control
F1NPA9	RPS3	KH type-2 domain-containing protein	2	4.13	0.0018	4.96	Control
A0A1D5P0E2	SEC23A	Protein transport protein SEC23	2	6.21	0.0022	1.53	Control
Q6SVA6	DMRT1	DMRT1 isoform e (Fragment)	2	4.38	0.0036	5.02	Control
A0A3Q2TUH0	PDXK	Phos_pyr_kin domain-containing protein	2	7.63	0.0047	2.05	Control
A0A1D5PMT8	RPLP2	Ribosomal protein lateral stalk subunit P2	2	6.88	0.0052	2.76	Control
A0A1D5P3J8	SHMT1	Serine hydroxymethyltransferase	2	6.51	0.0084	1.65	Control
A0A1L1RNL7	SEC13	WD_REPEATS_REGION domain-containing protein	2	5.48	0.0099	1.70	Control
Q90864	HBE1	Beta-H globin	2	5.93	0.0113	2.27	Control
F1NW23	CLTC	Clathrin heavy chain	2	6.51	0.0144	3.50	Control
A0A1D5PNR4	PRDX3	Peroxiredoxin 3	2	5.12	0.0147	19.17	Control
A0A1D5NYX6	CYB5A	Cytochrome B5 type A	2	7.25	0.0149	2.96	Control
R4GGJ0	RPS16	Ribosomal protein S16	2	4.66	0.0165	3.15	Control
A0A3Q2UED1	SEC31A	Protein transport protein Sec31A	2	3.96	0.0186	2.44	Control
A0A1D5PXH1	HNRNPA3	Heterogeneous nuclear ribonucleoprotein A3	2	6.09	0.0251	1.55	Control
A0A1L1RJP8	TUBA4A	Tubulin alpha 4a	2	6.59	0.0298	1.72	Control
E1BTG1	RPL12	60S ribosomal protein L12	2	4.92	0.0330	4.13	Control
F6T197	VDAC2	Voltage-dependent anion-selective channel protein 2 isoform X1	2	4.98	0.0337	3.89	Control
E1BV75	DHRS7	Dehydrogenase/reductase 7	2	5.57	0.0351	3.96	Control
E1C3Y2	HGD	Homogentisate 1,2-dioxygenase	2	7.53	0.0356	1.67	Control
A0A1D5P648	RDH16	Retinol dehydrogenase 16	2	5.61	0.0402	3.96	Control
Q8QH01	FMO3	Dimethylaniline monooxygenase [N-oxide-forming]	2	4.94	0.0405	8.16	Control
Q90WR6	SULT1C	Sulfotransferase family 1C	2	6.00	0.0481	4.01	Control

Fifty one proteins up-regulated in birds reared under Control lighting among 81 with altered levels compared to Cool LED lightings, identified by label-free LC-MS/MS (Progenesis QI for proteomics).

^a^) Accession number in the UniProt database. ^b^) Peptides used for quantitation. ^c^) SEQUEST score. ^d^) Indicates if the proteins were up-regulated in the birds reared with Control lighting.

In the comparison of broilers raised with Warm LED lighting and the Control lighting, eight proteins (from 21 peptides) were identified as differentially expressed. Among these, five proteins (62.5%) were up-regulated in broilers raised with Warm LED lighting, while three proteins (37.5%) were up-regulated in those raised with the Control lighting, as illustrated in Table 4.

**Table 4:** Birds reared with Warm LED lightings compared to birds reared with Control lighting.

UniProt^a^	Gene Name	Description	Peptides^b^	Score^c^	ANOVA (p)	Fold change	Highest condition^d^
A0A1D5PKN8	MPST	Mercaptopyruvate sulfurtransferase	3	8.61	0.010	1.76	Warm LED lightings
Q90892	GBP	Guanylate binding protein	3	8.13	0.034	5.61	Warm LED lightings
E1BXG9	PPID	Peptidylprolyl isomerase D	2	6.65	0.003	1.72	Warm LED lightings
A0A1D5PPN9	HSP90B1	Endoplasmin	2	5.98	0.005	289.62	Warm LED lightings
E1BSV1	DMGDH	Dimethylglycine dehydrogenase	2	4.45	0.039	10.74	Warm LED lightings
A0A1D5PSE5	ACLY	ATP-citrate synthase	4	10.56	0.024	1.79	Control
F1NT20	ACAT2	Acetyl-CoA acetyltransferase 2	3	7.78	0.014	2.26	Control
A0A3Q2UM65	RRBP1	Ribosome-binding protein 1	2	4.92	0.008	1.89	Control

Eight proteins identified with altered levels in response to Warm LED lightings compared to birds reared with Control lighting following label-free LC-MS/MS analysis (Progenesis QI for proteomics). Five proteins were up-regulated in the birds reared with Warm LED lightings and three proteins were up-regulated in the birds reared with Control lighting.

^a^) Accession number in the UniProt database. ^b^) Peptides used for quantitation. ^c^) SEQUEST score. ^d^) Indicates if the proteins were up-regulated in the birds reared with Warm LED lightings or with Control lighting.

**Fig 2:**
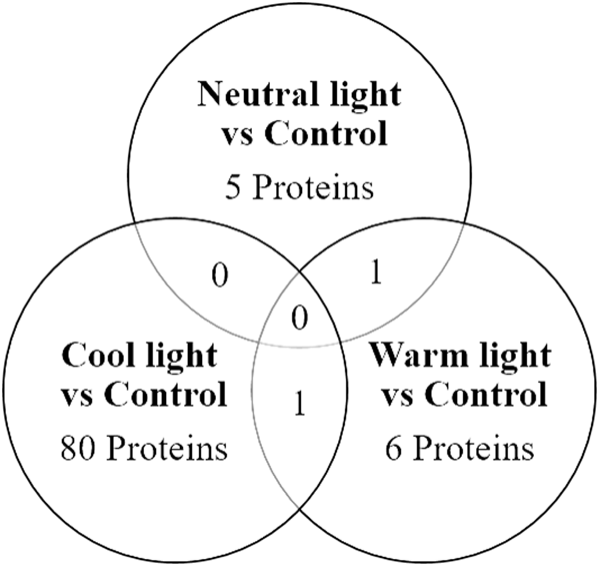
Venn diagram of the different shade of LED lighting compared to Control.

Fig 2 displays a Venn diagram illustrating the proteins that were common among those significantly different in the three comparisons. The diagram reveals that five, eighty, and six proteins identified in broilers raised in the comparisons with Neutral, Cool, or Warm LED lightings were unique to each respective comparison. Notably, no proteins were found to be common across all three comparisons. Specifically, one protein (Ribo-some-binding protein 1) was shared between the Neutral and Warm LED lighting comparisons, and another protein (ATP-citrate synthase) was common between the Cool and Warm LED lighting comparisons. Interestingly, there were no proteins identified as common between the Neutral and Cool LED lighting comparisons.

Venn diagrams showing the number of identified proteins with altered levels in response to the different shade of LED lighting (Neutral, Warm, and Cool) compared to Control. The full lists of proteins and the indication if they are up or down regulate are in Tables 1–4.

### Functional annotation of significantly different proteins

**Fig 3:**
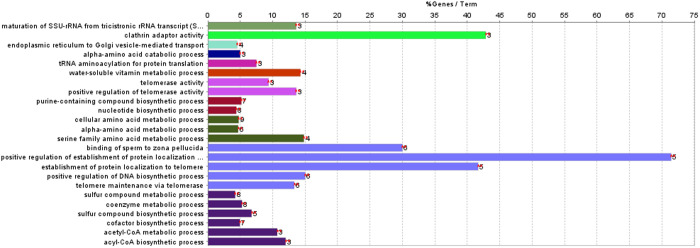
Gene enrichment analysis (Gene ontology-Biological process) of the 81 identified proteins (Cool LED lighting versus Control). The bars represent the percentage of input proteins associated with each functional term relative to the total number of directly annotated proteins. Next to each bar, the number of input proteins linked to that term is indicated. Bars with the same color are grouped into the same functional category (see S4 Table for details).

GO and KEGG pathway annotations were used to functionally characterize the proteins that were differentially expressed in response to the different shades of LED lighting (Neutral, Warm, and Cool) compared to the Control. Due to the low number of proteins identified as differentially expressed in response to Neutral LED lighting compared to the Control and in response to Warm LED lighting compared to the Control, no GO Terms were found. However, in the comparison where Cool LED lighting was compared to the Control, 24 GO:BP terms corresponding to 37 differentially abundant proteins were retrieved (see S4 Table, Fig 3). The most prevalent biological processes included positive regulation of establishment of protein (71.43%), clathrin adaptor activity (42.86%), and the establishment of protein localization to telomere (41.67%).

**Fig 4:**
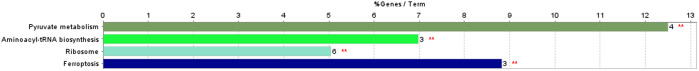
Gene enrichment analysis (KEGG pathway database) of the 81 identified proteins (Cool LED lighting versus Control). Each bar represents the percentage of input proteins associated with a specific functional term relative to the total number of directly annotated proteins. The number of input proteins for each term is shown next to the corresponding bar (see S5 Table for details).

A separate KEGG pathway analysis was performed on all differentially identified proteins in the chicken liver exudate. Also, for this analysis, no GO Terms were found for the comparisons involving Neutral and Warm LED lighting compared to Control. In the comparison where Cool LED lighting was compared to Control, four KEGG pathways were identified, involving 16 proteins. These proteins were associated with pyruvate metabolism (12.5%), ferroptosis (8.82%), minoacyl-tRNA biosynthesis (6,98%) and ribosome (5,04%), as presented in S5 Table and Fig 4.

### Protein protein interaction (PPI) analysis

**Fig 5:**
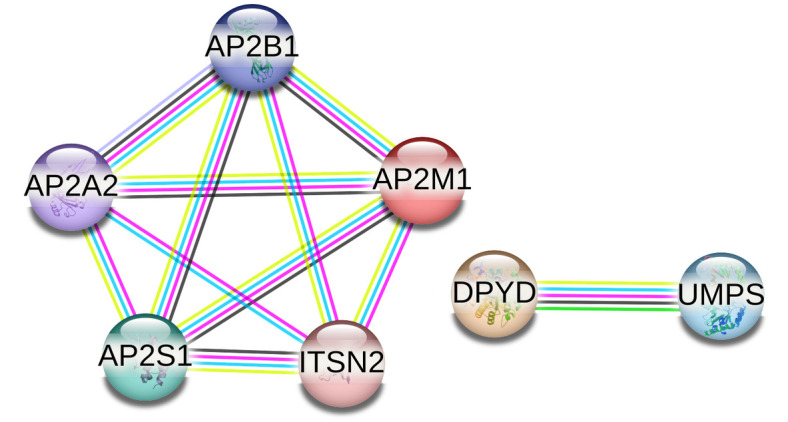
PPI network of the six proteins identified in the Neutral LED lighting versus Control comparison. In this network, each node represents a protein. The colored lines indicate the type of association: magenta for experimentally determined interactions; dark green for gene neighborhood; cyan for data from curated databases; black for coexpression; purple for protein homology; light green for text mining; and blue for gene co-occurrence.

The STRING database was used to identify functional interactions among proteins that were differentially expressed in response to various LED lighting conditions (Neutral, Warm, and Cool) compared to the Control. In the comparison between broilers raised with Neutral LED lighting and the Control (six proteins), the analysis revealed a connected protein network (Fig 5) divided into two components: i) one module composed of five nodes (71.43%), and ii) one component of two proteins (28.57%). The PPI enrichment had a *p-*value of 0.0184 (with 5 expected edges vs. 11 detected edges), indicating that the proteins are at least partially biologically connected. The majority of the proteins in this network interacted with two other partners (average node degree equal to 2).

**Fig 6:**
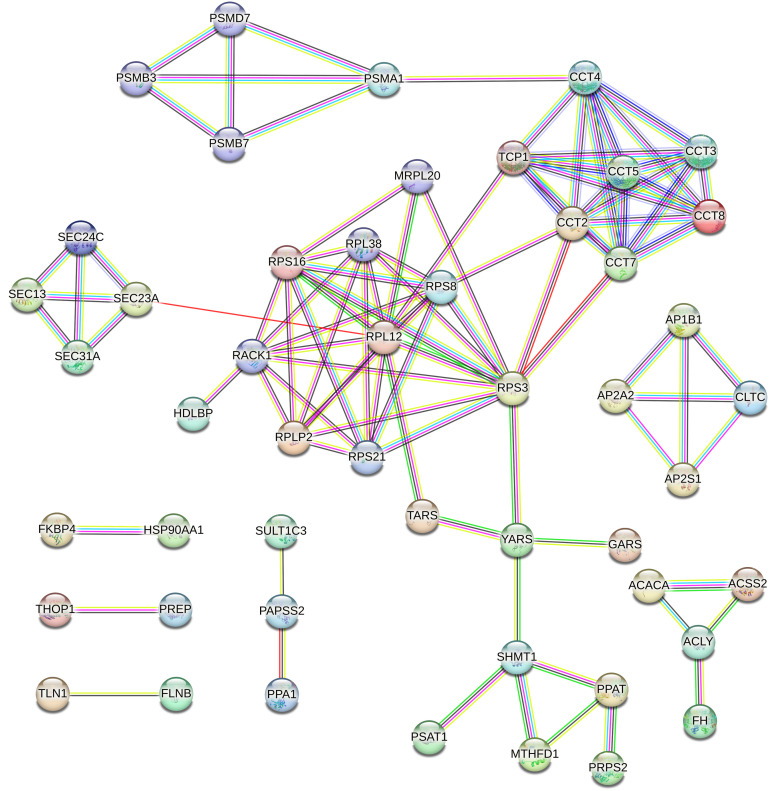
PPI network of the 81 identified proteins in the Cool LED lighting versus Control comparison. In this network, each node represents a protein. The colored lines indicate the type of association: magenta for experimentally determined interactions; dark green for gene neighborhood; cyan for data from curated databases; black for coexpression; purple for protein homology; light green for text mining; and blue for gene co-occurrence.

The analysis of differentially expressed proteins (81 proteins) between broilers raised with Cool LED lighting and the Control uncovered a connected protein network (Fig 6) consisting of: i) one large module with 33 nodes (66%), ii) two modules with 4 nodes (16%), iii) one small module with three nodes (6%), and iv) three small components with two proteins (12%), totaling 50 nodes. The PPI enrichment resulted in a *p*-value of <1.0e-16 (34 expected edges vs. 96 detected edges), indicating that proteins are at least partially biologically connected. Notably, the majority of proteins in this network interacted with two or three other partners, with an average node degree of 2.31.

**Fig 7:**
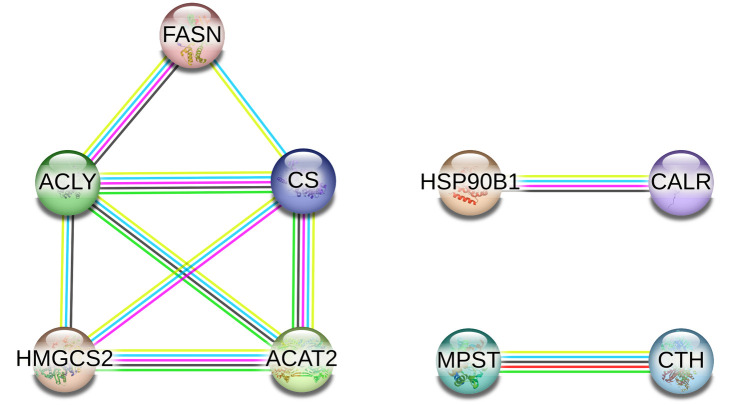
PPI network of the eight identified proteins in the Warm LED lighting versus Control comparison. In this network, each node represents a protein. The colored lines indicate the type of association: magenta for experimentally determined interactions; dark green for gene neighborhood; cyan for data from curated databases; black for coexpression; purple for protein homology; light green for text mining; and blue for gene co-occurrence.

In the comparison of broilers raised with Warm LED lighting and the Control (eight proteins), the analysis revealed a connected protein network (Fig 7), which was divided into the following components: i) one module of five nodes (55.55%) and ii) two small components of two proteins (44.45%). The PPI enrichment had a *p*-value of 0.0475 (with 5 expected edges vs. 10 detected edges), indicating that the proteins are at least partially biologically connected. The majority of the proteins in this network interacted with one or two other partners (average node degree equal to 1.67).

## Discussion

Light is a key environmental factor that significantly impacts the physiology, behavior, immunity, and growth performance of chickens [3,4]. Poultry heavily rely on visual cues to identify safe food and water sources, and their appropriate feeding behaviour is facilitated by an innate inclination to peck at small particles and flat, shiny surfaces. Furthermore, vision is likely the predominant sense in domestic poultry, given that the majority of their behavior is influenced by sight [39,40]. Numerous studies have explored the effects of different light sources on the welfare and growth performance of broilers [1,11,13,14]. However, achieving optimal production necessitates research that investigates how light impacts the biochemical and molecular processes in birds. In the present study, we addressed this issue by applying label-free proteomics profiling to analyze liver protein abundance patterns in broilers reared under various light sources, including three shades of LED lights (Neutral, Cool, and Warm) and Control lighting. Our goal was to uncover peptides and proteins indicative of biological responses to these lighting conditions.

To our knowledge, this is the first study that applies a proteomics method to elucidate the influence of different light sources on the chicken proteome. In our study, the label-free LC-MS analysis revealed a similar number of proteins (average: 1181 proteins) in the three comparisons under study (shades of LED lights Neutral, Cool, and Warm versus Control lighting). The proteins in all comparisons were found to be involved in similar biological processes such as cellular processes, metabolic processes, biological regulation, localization, and response to stimulus.

Among proteomics studies, differences in the number of proteins identified can be observed even when a similar matrix is used. These differences may arise from various factors, such as different methods of protein extraction employed (e.g., exudate, homogenized), various methods of protein purification (e.g., filter-aided sample preparation (FASP), in-StageTip1 (iST)), mass spectrometry methods utilized for peptide and protein identification, and the database utilized. Each approach has its own advantages and disadvantages, which determine the number of detected peptides/proteins and the quality of the data obtained. For example, studies applying a proteomics approach similar to our study on the chicken liver proteome to investigate the effects of immune stress on the hepatic proteome and heat stress identified a higher number of proteins [27,41], whereas a study comparing the liver proteome among different pig breeds identified a lower number of proteins [28]. In our study, liver exudate collected following centrifugation of liver specimens was used as the substrate. The advantage of this method of protein extraction is its simplicity, allowing the obtention of a substrate that is rich in proteins. In our method, we utilized a low speed of centrifugation so that this speed could be achieved with any centrifuge and thus applicable as starting material in any environment, such as an abattoir. Moreover, the reduced handling of the protocol allows for the obtention of a substrate that is highly reproducible, producing a high-quality matrix for sample preparation for proteomic analysis [42].

Different findings have been found regarding the effects of these LED light bulbs on broiler performance, potentially stemming from variations in the specific bulbs and their quality across different manufacturers [4,15,43]. A previously conducted study in our laboratory using the same batches of birds employed in this study indicated that the Warm LED lighting negatively influenced the live weight and carcass weight of broilers compared to the Control group (neon lighting), while no significant changes were observed for Neutral and Cool LED lighting. However, in that same study, high drip loss was observed in breast meat samples obtained from broilers reared under Neutral and Cool LED lighting compared to the Control. No significant differences were observed in carcass yield and cooking loss under any conditions. Overall, despite the observed increase in drip loss under certain LED conditions, the study concluded that the use of LED lights in broiler breeding remains a viable alternative to traditional neon lighting, as it does not negatively impact core production parameters and only minimally affects specific aspects of meat quality without compromising overall product acceptability. Additionally, it is more sustainable from both an economic and environmental standpoint [30].

In our study, using LC-MS/MS profiling, a small difference in the proteome between the Control and Neutral lighting (six proteins) and between the Control and Warm lighting (eight proteins) was observed. However, 81 proteins involved in several biological processes were found to be differentially expressed between the Control and Cool lighting. These data indicate that the variations observed among the three different light comparisons may be linked to various aspects of the proteome, involving a range of molecular functions. The differences observed at the protein level between the different light comparisons are attributed to phenotypic plasticity, which involves modulation of gene expression and downstream protein synthesis in response to the environment [44]. This process enables a more effective adaptation of the bird to the different shade of lights, indicating that in our study, a greater molecular adaptation of the birds was observed in the broiler housed with Cool LED lighting, whereas less pronounced molecular adjustments were observed in response to the Neutral and Warm LED lighting.

Ribosome-binding protein 1 (RRBP1) is an endoplasmic reticulum (ER) membrane-associated protein that plays a critical role in anchoring ribosomes to the ER and facilitating the translocation and secretion of newly synthesized proteins in eukaryotic cells [45]. Studies in yeast have shown that RRBP1 is involved in the endoplasmic reticulum stress response and the associated unfolded protein response, both of which are critical for signaling pathways originating from the endoplasmic reticulum [46]. It has been suggested that RRBP1 may be a key molecule in the signaling network of endoplasmic reticulum stress, the unfolded protein response, and autophagy [47,48]. Additionally, RRBP1 has been identified as a potential protein marker for colorectal cancer [49]. In our study, RRBP1 was up-regulated in birds exposed to Control lighting compared to those exposed to both Neutral and Warm LED lighting. This suggests that birds housed under Control lighting experience slightly higher stress levels (fold change: 1.76/1.89) compared to those under Neutral and Warm LED lighting, indicating an adaptive role of RRBP1 to stress conditions. A comparable up-regulation of RRBP1 protein expression has been reported in tumor cells, where it has been linked to elevated mRNA levels resulting from gene amplification and/or increased transcriptional activity [50,51].

ATP-citrate lyase (ACLY) was found to be up-regulated in birds exposed to Control lighting compared to those exposed to both Cool and Warm LED lighting. Citrate synthase, an enzyme present in nearly all cells capable of oxidative metabolism, catalyzes the condensation of acetyl-CoA and oxaloacetate, releasing coenzyme A and citric acid. It serves as a rate-limiting enzyme in the mitochondrial tricarboxylic acid (TCA) cycle, playing a crucial role in numerous biochemical processes [52]. The activity of citrate synthase within the TCA cycle is inhibited by succinyl-CoA, but remains unaffected by acetyl-CoA or oxaloacetate [53]. ACLY also participates in the regulation of various biological processes, including the respiratory quotient, mitochondrial energy expenditure, lymphocyte growth (as demonstrated in mammalian models), and the progression of certain diseases [54]. High expression of citrate synthase enhances the TCA cycle, thereby increasing the availability of reducing equivalents (NADH, FADH₂) that fuel the electron transport chain, ultimately supporting the activity of ATP synthase complexes and boosting ATP production and energy metabolism [55,56]. This expression may be triggered by a cascade of events initiated by the release of large amounts of adrenocorticotropic hormone from the adenohypophysis when animals are exposed to various harmful stimuli, such as trauma, infection, cold, fear. In our study, the proteomic changes observed in broilers exposed to neon lighting suggest that certain artificial lighting conditions may also act as environmental stressors capable of eliciting similar endocrine responses [56–58]. This process could play a significant role in improving the bird’s tolerance to harmful stimuli, thereby reducing adverse reactions and maintaining physiological function.

Several ribosomal proteins were up-regulated in birds exposed to Control lighting compared to those exposed to Cool LED lighting. Ribosomes are complex molecular machines composed of four ribosomal RNAs and around 80 ribosomal proteins. In eukaryotes, they consist of two subunits: the 40S small subunit, responsible for recognizing and binding mRNA, and the 60S large subunit, which facilitates peptide bond formation. Together, these subunits form the 80S ribosome, which plays a central role in translating mRNA into proteins [59,60]. Beyond its primary function of protein synthesis, the ribosome influences key cellular processes such as proliferation, differentiation, apoptosis, and transformation [61,62]. Disruptions in ribosome biogenesis or function can result in cellular dysfunction and contribute to various diseases, including cardiovascular disorders, neurodegenerative conditions, cancer, viral infections, and bacterial resistance [63–65].

In our study, several ribosomal proteins, including those involved in the 40S and 60S subunits, were up-regulated in birds under Control lighting, suggesting increased ribosome activity in these conditions. This is consistent with previous findings showing that ribosomal dysfunction can effect cell fate. For example, Albert et al. [66] found that when ribosome biogenesis was blocked in *Saccharomyces cerevisiae*, it triggers a stress response that increased heat shock genes expression and reduced ribosomal protein genes expression. Similarly, in our study, birds raised under Cool LED lighting showed lower expression of ribosomal proteins in the liver, suggesting that Cool LED lighting may act as a stronger stressor compared to neon light.

Although this is speculative, it may help explain why more differentially expressed proteins were found under Cool LED lighting (81 proteins) than under Neutral and Warm LED lighting (six and eight proteins, respectively). This reduction in ribosomal activity under stress may reflect a physiological adaptation specific to cooler LED lighting conditions.

This hypothesis is further supported by the up-regulation of several proteins involved in aminoacyl-tRNA biosynthesis, such as glycine--tRNA ligase (GARS), tyrosine--tRNA ligase (YARS), and the AA_TRNA_LIGASE_II domain-containing protein (TARS), in birds exposed to Cool LED lighting. Aminoacyl-tRNA synthesis is a critical cellular process that supplies substrates for ribosomal translation of mRNA during protein synthesis. This process plays a central role in bridging nucleic acid sequences with their corresponding amino acids, ensuring the accurate transmission of genetic information [67]. The specificity of aminoacyl-tRNA synthesis in matching the correct tRNAs with their respective amino acids is crucial for maintaining fidelity in protein translation; errors in this process can result in faulty protein synthesis [68].

Interestingly, the up-regulation of the aminoacyl-tRNA biosynthesis pathway has been reported in several types of cancers, including gastric cancer, pancreatic adenocarcinoma, clear cell renal cell carcinoma, prostate adenocarcinoma, diffuse large B-cell lymphoma, and the follicular variant of papillary thyroid carcinoma [69–73]. Proteins such as TARS, LARS, and YARS, identified in our study, have been correlated to these cancers, with TARS, in particular, associated with tumor metastasis and cell proliferation [69,74]. In our study, these proteins were up-regulated in the liver of birds exposed to Cool LED lighting, suggesting that this lighting condition may be a stronger stressor for the birds than the other tested. However, these findings reflect molecular-level changes in the liver proteome and do not necessarily correlate with the physical and chemical properties of breast meat reported in reference [30]. This underscores the need to evaluate both molecular responses and product quality perspectives when assessing lighting conditions.

## Conclusions

In conclusion, although this work is a pilot study and further validation using additional methods and a larger sample size is needed, our label-free quantitative proteomics analysis identified significant differences in liver protein abundance patterns in broilers reared under different lighting conditions. In particular, the three LED lighting conditions (Neutral, Cool, and Warm) showed distinct proteomic profiles compared to Control lighting. Minimal proteomic changes were observed between the Control and Neutral lighting (six proteins) and between the Control and Warm lighting (eight proteins). However, a striking difference was noted with Cool LED lighting, where 81 proteins involved in various biological processes were found to be differentially expressed. This suggests that Cool LED lighting may induces adaptive response in broilers and potentially acts as a greater stressor compared to Control lighting. Consequently, our findings suggest that rearing birds under Cool LED lighting throughout their entire life cycle may induce stress in the liver, whereas Neutral and Warm LED lighting are more favorable for maintaining liver health. These observations emphasize the potential for physiological adaptation under Cool LED lighting, even though previous findings (reference [30]) showed no significant impact on meat quality parameters. Additionally, Neutral and Warm LED lighting may also provide economic and environmental advantages, making them more sustainable lighting solutions for poultry farming.

## Supporting information

S1 Table Complete list of the 1170 proteins identified by mass spectrometry in broilers reared under Neutral LED lighting and Control lighting. (XLSX)

S2 Table Complete list of the 1141 proteins identified by mass spectrometry in broilers reared under Cool LED lighting and Control lighting. (XLSX)

S3 Table Complete list of the 1231 proteins identified by mass spectrometry in broilers reared under Warm LED lighting and Control lighting. (XLSX)

S4 Table Over-represented Biological Processes (GO:BP) associated with proteins that are either up- or down-regulated in chickens reared under Cool LED lighting compared to Control lighting. ^1^Groups of closely related terms are shown; ^2^Benjamini–Hochberg corrected p-values are provided; ^3^The percentage of input proteins associated with each term is reported relative to the total number of proteins directly annotated with that term; ^4^The symbol ↑ indicates higher protein abundance in chickens reared under Cool LED lighting, while ↓ indicates higher abundance in chickens reared under Control lighting.
(DOCX)

S5 Table Over-represented KEGG pathways associated with proteins that are either up- or down-regulated in chickens reared under Cool LED lighting compared to Control lighting. ^1^Groups of closely related terms are shown; ^2^Benjamini–Hochberg corrected p-values are provided; ^3^The percentage of input proteins associated with each pathway is reported relative to the total number of proteins directly annotated with that term; ^4^The symbol ↑ indicates higher protein abundance in chickens reared under Cool LED lighting, while ↓ indicates higher abundance in chickens reared under Control lighting.
(DOCX)
