# Supplementary material for: Proteomic insights into broiler stress responses to LED lighting: Effects on liver proteome under neutral, cool, and warm spectra
Source: PLoS One. 2025 Jul 15;20(7):e0328279. doi: 10.1371/journal.pone.0328279 (PMC12262841; doi:10.1371/journal.pone.0328279)
Supplement: S4 Table — 1Groups of closely related terms are shown; 2Benjamini–Hochberg corrected p-values are provided; 3The percentage of input proteins associated with each term is reported relative to the total number of proteins directly annotated with that term; 4The symbol ↑ indicates higher protein abundance in chickens reared under Cool LED lighting, while ↓ indicates higher abundance in chickens reared under Control lighting. (DOCX) [file pone.0328279.s004.docx]

**Table S4.** Over-represented Biological Processes (GO:BP) associated with proteins that are either up- or down-regulated in chickens reared under Cool LED lighting compared to Control lighting.

| GOID | Description | Functional  group^1^ | pvalue^2^ | % of associated  proteins^3^ | N. of  proteins | Up or down regulated proteins ^4^ |
| --- | --- | --- | --- | --- | --- | --- |
| GO:0000462 | maturation of SSU-rRNA from tricistronic rRNA transcript (SSU-rRNA, 5.8S rRNA, LSU-rRNA) | G0 | 0,000445 | 13,64 | 3 | [RPS16↓, RPS21↓, RPS8↓] |
| GO:0035615 | clathrin adaptor activity | G1 | 0,000019 | 42,86 | 3 | [AP2A2↓, CLAPS2↓, TLN1↑] |
| GO:0006888 | endoplasmic reticulum to Golgi vesicle-mediated transport | G2 | 0,001994 | 4,55 | 4 | [SEC13↓, SEC23A↓, SEC24C↓, SEC31A↓] |
| GO:1901606 | alpha-amino acid catabolic process | G3 | 0,005355 | 5,00 | 3 | [HAL↓, HGD↓, SHMT1↓] |
| GO:0006418 | tRNA aminoacylation for protein translation | G4 | 0,002056 | 7,50 | 3 | [GARS↑, TARS↑, YARS↑] |
| GO:0006767 | water-soluble vitamin metabolic process | G5 | 0,000038 | 14,29 | 4 | [GCLC↑, HAGH↑, PDXK↓, SHMT1↓] |
| GO:0003720 | telomerase activity | G6 | 0,001201 | 9,38 | 3 | [CCT2↓, HSP90AA1↑, TCP1↓] |
| GO:0051973 | positive regulation of telomerase activity | G6 | 0,001201 | 13,64 | 3 | [CCT2↓, HSP90AA1↑, TCP1↓] |
| GO:0072522 | purine-containing compound biosynthetic process | G7 | 0,000033 | 5,19 | 7 | [ACACA↓, ACLY↓, ACSS2↑, MTHFD1↓, PRPS2↓, SHMT1↓, VDAC2↓] |
| GO:0009165 | nucleotide biosynthetic process | G7 | 0,000033 | 4,40 | 8 | [ACACA↓, ACLY↓, ACSS2↑, GARS↑, MTHFD1↓, PRPS2↓, SHMT1↓, VDAC2↓] |
| GO:0006520 | cellular amino acid metabolic process | G8 | 0,000009 | 4,76 | 9 | [GARS↑, GCLC↑, HAL↓, HGD↓, MTHFD1↓, PSAT1↓, SHMT1↓, TARS↑, YARS↑] |
| GO:1901605 | alpha-amino acid metabolic process | G8 | 0,000009 | 4,69 | 6 | [GCLC↑, HAL↓, HGD↓, MTHFD1↓, PSAT1↓, SHMT1↓] |
| GO:0009069 | serine family amino acid metabolic process | G8 | 0,000009 | 14,81 | 4 | [GCLC↑, MTHFD1↓, PSAT1↓, SHMT1↓] |
| GO:0007339 | binding of sperm to zona pellucida | G9 | 0,000004 | 30,00 | 6 | [CCT2↓, CCT5↓, CCT7↓, CCT8↓, TCP1↓, VDAC2↓] |
| GO:1904851 | positive regulation of establishment of protein localization to telomere | G9 | 0,000004 | 71,43 | 5 | [CCT2↓, CCT5↓, CCT7↓, CCT8↓, TCP1↓] |
| GO:0070200 | establishment of protein localization to telomere | G9 | 0,000004 | 41,67 | 5 | [CCT2↓, CCT5↓, CCT7↓, CCT8↓, TCP1↓] |
| GO:2000573 | positive regulation of DNA biosynthetic process | G9 | 0,000004 | 15,00 | 6 | [CCT2↓, CCT5↓, CCT7↓, CCT8↓, HSP90AA1↑, TCP1↓] |
| GO:0007004 | telomere maintenance via telomerase | G9 | 0,000004 | 13,33 | 6 | [CCT2↓, CCT5↓, CCT7↓, CCT8↓, HSP90AA1↑, TCP1↓] |
| GO:0006790 | sulfur compound metabolic process | G10 | 0,000022 | 4,23 | 8 | [ACACA↓, ACLY↓, ACSS2↑, GCLC↑, HAGH↑, MTHFD1↓, PAPSS2↓, SULT1C3↓] |
| GO:0006732 | coenzyme metabolic process | G10 | 0,000022 | 5,26 | 8 | [ACACA↓, ACLY↓, ACSS2↑, GCLC↑, HAGH↑, MTHFD1↓, PDXK↓, SHMT1↓] |
| GO:0044272 | sulfur compound biosynthetic process | G10 | 0,000022 | 6,76 | 5 | [ACACA↓, ACLY↓, ACSS2↑, GCLC↑, HAGH↑] |
| GO:0051188 | cofactor biosynthetic process | G10 | 0,000022 | 4,93 | 7 | [ACACA↓, ACLY↓, ACSS2↑, GCLC↑, HAGH↑, MTHFD1↓, PDXK↓] |
| GO:0006084 | acetyl-CoA metabolic process | G10 | 0,000022 | 10,71 | 3 | [ACACA↓, ACLY↓, ACSS2↑] |
| GO:0071616 | acyl-CoA biosynthetic process | G10 | 0,000022 | 12,00 | 3 | [ACACA↓, ACLY↓, ACSS2↑] |

^1)^ Groups of closely related terms are shown.

^2)^ Benjamini–Hochberg corrected p-values are provided.

^3)^ The percentage of input proteins associated with each term is reported relative to the total number of proteins directly annotated with that term.

^4)^ The symbol ↑ indicates higher protein abundance in chickens reared under Cool LED lighting, while ↓ indicates higher abundance in chickens reared under Control lighting.
