# Supplementary material for: Proteomic insights into broiler stress responses to LED lighting: Effects on liver proteome under neutral, cool, and warm spectra
Source: PLoS One. 2025 Jul 15;20(7):e0328279. doi: 10.1371/journal.pone.0328279 (PMC12262841; doi:10.1371/journal.pone.0328279)
Supplement: S5 Table — 1Groups of closely related terms are shown; 2Benjamini–Hochberg corrected p-values are provided; 3The percentage of input proteins associated with each pathway is reported relative to the total number of proteins directly annotated with that term; 4The symbol ↑ indicates higher protein abundance in chickens reared under Cool LED lighting, while ↓ indicates higher abundance in chickens reared under Control lighting. (DOCX) [file pone.0328279.s005.docx]

**Table S5.** Over-represented KEGG pathways associated with proteins that are either up- or down-regulated in chickens reared under Cool LED lighting compared to Control lighting.

| GOID | Description | Functional  group^1^ | pvalue^2^ | % of associated  proteins^3^ | N. of  proteins | Up or down regulated proteins ^4^ |
| --- | --- | --- | --- | --- | --- | --- |
| KEGG:00620 | Pyruvate metabolism | G0 | 0,001 | 12,50 | 4 | [ACACA↓, ACSS2↑, FH↑, HAGH↑] |
| KEGG:00970 | Aminoacyl-tRNA biosynthesis | G1 | 0,008 | 6,98 | 3 | [GARS↑, TARS↑, YARS↑] |
| KEGG:03010 | Ribosome | G2 | 0,002 | 5,04 | 6 | [RPL12↓, RPLP2↓, RPS16↓, RPS21↓, RPS3↓, RPS8↓] |
| KEGG:04216 | Ferroptosis | G3 | 0,005 | 8,82 | 3 | [ACSL5↓, GCLC↑, VDAC2↓] |

^1)^ Groups of closely related terms are shown.

^2)^ Benjamini–Hochberg corrected p-values are provided.

^3)^ The percentage of input proteins associated with each pathway is reported relative to the total number of proteins directly annotated with that term.

^4)^ The symbol ↑ indicates higher protein abundance in chickens reared under Cool LED lighting, while ↓ indicates higher abundance in chickens reared under Control lighting.
